# Occupational therapist‐guided exercise increased white blood cell and neutrophil counts during clozapine treatment: A case report

**DOI:** 10.1002/pcn5.70167

**Published:** 2025-07-28

**Authors:** Kenji Hinotsu, Shinji Sakamoto, Hiroki Kawai, Yoshio Ohya, Akiyoshi Yokode, Takahiro Asada, Yuko Okahisa, Manabu Takaki

**Affiliations:** ^1^ Department of Neuropsychiatry Okayama University Hospital Okayama Japan; ^2^ Department of Neuropsychiatry Okayama University Faculty of Medicine, Dentistry and Pharmaceutical Sciences Okayama Japan; ^3^ Department of Neuropsychiatry Okayama University Graduate School of Medicine, Dentistry and Pharmaceutical Sciences Okayama Japan

**Keywords:** clozapine, exercise, leukopenia, neutropenia, occupational therapist

## Abstract

**Background:**

Moderate exercise increases white blood cells and neutrophils. However, there are no reports on the relationship between exercise intensity and these cells. We observed a patient taking clozapine whose white blood cell and neutrophil counts were borderline. Supervised exercise therapy with an occupational therapist stabilized these counts.

**Case Presentation:**

A 50‐year‐old woman with treatment‐resistant schizophrenia was prescribed clozapine. By Day 63, the clozapine dosage had been increased to 450 mg/day. Additionally, she was advised to perform a 30‐min walking exercise program 1 h before blood tests. Exercise therapy supervised by an occupational therapist was performed eight times, and self‐training was performed five times. Exercise intensity was monitored using the Borg Scale for subjective evaluation and the Karvonen formula for objective evaluation. Supervised exercise therapy with an occupational therapist resulted in greater increases on the Borg Scale and Karvonen formula than did self‐training. It also induced increases in white blood cells and neutrophils. Her psychiatric symptoms improved, and she was discharged on Day 71. A blood test taken after discharge revealed that her white blood cell and neutrophil counts were within the normal range and she continued to take clozapine for 2 years. She has since been able to enjoy a calm and relaxed life at home.

**Conclusion:**

Exercise involving subjective and objective evaluation by an occupational therapist effectively increased white blood cells and neutrophils during clozapine treatment. Supervised exercise therapy by an occupational therapist is important when self‐exercise is insufficient for continuing clozapine treatment.

## BACKGROUND

Clozapine (CZP) is an antipsychotic medication for patients with treatment‐resistant schizophrenia (TRS).^1^ Due to the side‐effects, which include leukopenia and neutropenia,^2^ blood monitoring is required. The Japanese Clozaril Patient Monitoring Service (CPMS) is stricter than that in other countries.^3^ TRS patients in Japan need hospitalization, and a white blood cell (WBC) count of 4000/mm^3^ (3500/mm^3^ in other countries) is required at the initiation of CZP. At less than WBC 4000/mm^3^ or neutrophil (NE) 2000/mm^3^ (WBC: 3500/mm^3^ or NE: 2000/mm^3^ in other countries), TRS patients are required to take blood tests twice a week until the count returns to a normal range. In the case of a NE count of less than 2000/mm^3^, they continue to take a blood test every week for 26 weeks after it returns to normal range. In Japan, CZP is never administered if WBC counts drop to less than 3000/mm³ or NE counts drop to less than 1500/mm^3^.

Moderate exercise is known to enhance immune function and increase WBC and NE counts,^4^, ^5^ but there are no reports detailing the relationship between exercise intensity and WBC and NE counts. We had one patient who was administered CZP but whose WBC and NE counts were borderline for CPMS. Supervised exercise therapy with an occupational therapist (OT) stabilized the patient's WBC and NE counts.

## CASE PRESENTATION

Written informed consent has been obtained from the patient and her family to publish this report. A woman aged in her 50s first experienced auditory hallucinations and cognitive impairment in high school and was diagnosed with schizophrenia. Her symptoms were slightly alleviated by antipsychotic medications, including 12 mg/day of haloperidol for 4 months, 12 mg/day of bromperidol and 12 mg/day of risperidone for 13 years, and 20 mg/day of olanzapine for 2 years; however, her symptoms persisted. In her early 50s, she was hospitalized for the first time due to worsening auditory hallucinations and wandering. Communicating with her was difficult. She underwent six sessions of electroconvulsive therapy, which reduced her symptoms; however, the auditory hallucinations persisted. Based on her treatment history, she met the eligibility criteria for the TRS program as defined by the CPMS guidelines in Japan. Initiation of CZP was planned. However, because her WBC count was 3360/mm³ and her NE count was 1820/mm³, CZP could not be initiated. Adenine (60 mg/day), cepharanthine (6 mg/day), and lithium carbonate (600 mg/day) were administered for 19 days to increase the WBC and NE levels. However, her WBC count varied, ranging from 3200/mm³ to 4800/mm³, and her NE count ranged from 1800/mm³ to 2900/mm³. Subsequently, adenine, cepharanthine, and lithium carbonate were discontinued. She was discharged and continued at the outpatient unit. Twenty months later, her WBC count was 5140/mm³ and her NE count was 4180/mm³.

**Figure 1:**
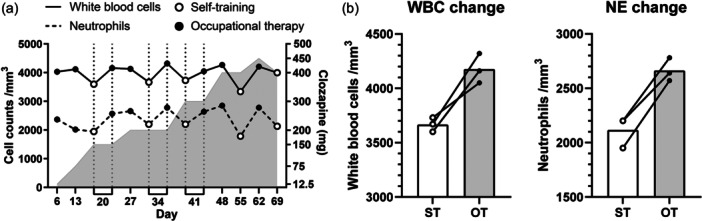
Clinical course. (a) The *x*‐axis indicates the number of days after hospitalization. The *y*‐axis shows cell counts, with white blood cells (WBCs) represented by a solid line and neutrophils (NEs) represented by a dotted line. Black dots on each line represent occupational therapy (OT), and white dots represent self‐training (ST), indicating post‐exercise cell counts. The right *y*‐axis shows the daily clozapine dosage as a bar chart. (b) The highlighted image shows WBC and NE counts when blood was sampled twice on the same day due to low counts. These figures demonstrate that, compared to self‐training (ST), occupational therapy (OT) leads to a more effective transition of WBC and NE counts to functional values. “Day” refers to the number of days after hospitalization.

**Figure 2:**
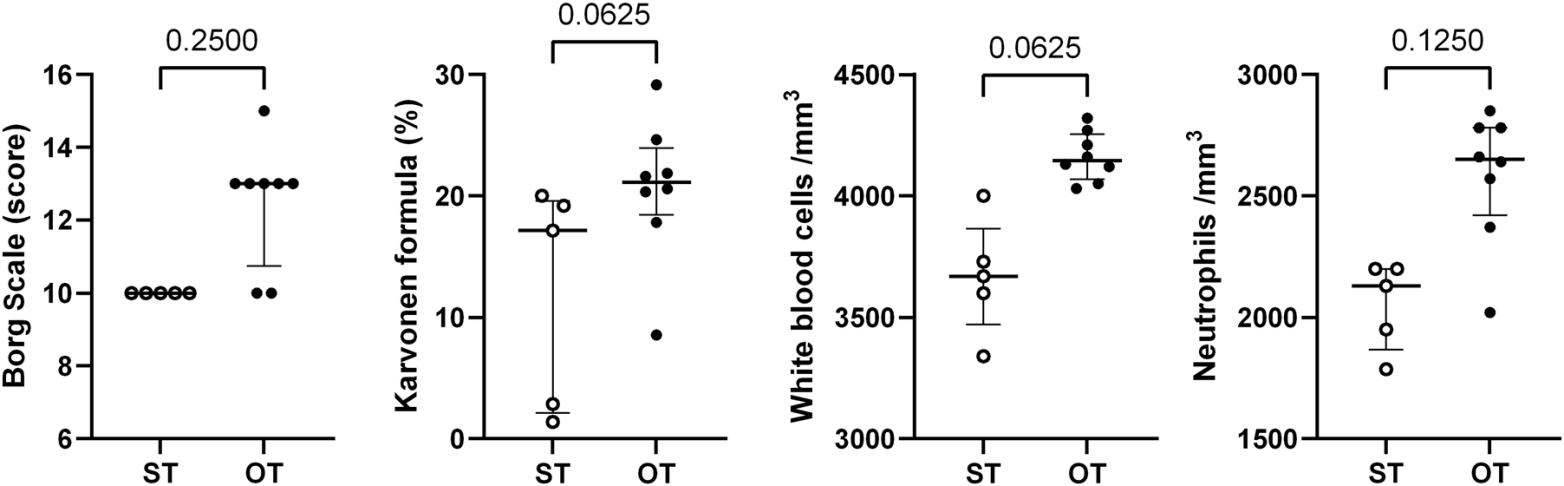
Scatter plots showing the relationship between self‐training (ST) and occupational therapy (OT) exercise. Each dot represents a single sample. Solid lines indicate the median and interquartile range (IQR). From left to right, the panels show the Borg Scale, the Karvonen formula, the white blood cell (WBC) count, and the neutrophil (NE) count. These differences between OT and ST were not statistically significant by the Wilcoxon signed‐rank test.

In response to her strong request, CZP was initiated. After being hospitalized, she was prescribed adenine (60 mg/day) and started taking cepharanthine (6 mg/day) again as a preventive measure. CZP was initiated at 12.5 mg/day 6 days after hospitalization and gradually increased. On Day 56, the CZP dose was titrated to 450 mg/day. Due to drowsiness and tremors, the CZP dosage was reduced to 400 mg/day on Day 63. Thirty minutes of walking exercise therapy was recommended before a blood test. The OT instructed her to pay attention to the increased intensity of her exercise, including her feeling of increased heart rate and fatigue, so that she could form the habit of exercising. Supervised exercise therapy with an OT occurred eight times, and self‐training (ST) occurred five times. Exercise intensity was monitored using the Borg Scale^6^ for subjective evaluation and the Karvonen formula^7^, ^8^ for objective evaluation. The Borg Scale was verbally confirmed by the OT, and the Karvonen formula was assessed using a pulse oximeter before and after exercise. Figure 1 shows the treatment timeline. We compared WBC and NE counts after OT or ST exercise using a standardized exercise scale (Figure 2). Borg Scale scores, pooled from the OT and ST groups, were significantly correlated with WBC counts (r² = 0.4113, *p* = 0.0182) and NE counts (r² = 0.592, *p* = 0.0021). The Karvonen formula scores, pooled from the OT and ST groups, were significantly correlated with NE counts (r² = 0.419, *p* = 0.0314) but not with WBC counts (r² = 0.145, *p* = 0.2479). The median (interquartile range [IQR]) Borg Scale score was 13 (12.3–13) for OT exercise and 10 (10–10) for ST exercise. The median (IQR) percentage of the Karvonen formula was 21.1 (19.7–22.6) for OT exercise and 17.1 (2.9–19.2) for ST exercise. The median (IQR) of WBCs was 4145 (4102.5–4225.0)/mm³ by OT exercise and 3670 (3600–3730)/mm³ by ST exercise. The median (IQR) of NEs was 2650 (2520–2780)/mm³ by OT exercise and 2130 (1950–2200)/mm³ by ST exercise. These differences between OT exercise and ST exercise were not statistically significant by the Wilcoxon signed‐rank test. The patient's psychiatric symptoms were reduced and her side‐effects diminished. She was discharged on Day 71. The OT instructed the patient to continue ST exercise based on the structured exercise therapy with a Borg Scale rating of 13 (“somewhat hard”). During her inpatient treatment, she developed the habit of doing exercises supervised by an OT. While receiving outpatient treatment, she continued doing these exercises at home every day. According to blood tests after discharge, her WBCs and NEs were within normal range and CZP was continued for 2 years. She has been able to live a calm and relaxed life at home. She has no history of smoking tobacco.

## DISCUSSION

First, we investigated the relationship between exercise intensity and WBC and NE in detail and found that exercise intensity correlates with WBC and NE. Although exercise is well known to increase WBC and NE counts, the intensity of the exercise is also important. The patient had a long history of schizophrenia and had been unable to work. Her willingness to exercise may also have been low due to the negativism associated with schizophrenia. The exercise intensity was evaluated as “very light” to “fairly light” for ST exercise and “somewhat hard” for OT exercise using the Borg Scale. The ST exercise during hospitalization was insufficient, and the OT's instruction was necessary to achieve an adequate intensity to increase WBCs or NEs. After discharge, the patient's WBC and NE counts remained stable. One reason for this is that she continued to exercise at an adequate intensity level (Borg Scale rating of 13) at home, as instructed by the OT during her hospitalization. Although opinions differ, CZP has been reported to improve negative symptoms, including cooperativeness.^9^ Another reason for the patient's WBC and NE counts remaining stable is that CZP improved her symptoms, including her willingness to exercise, and increased her activity at home.

Initially, the patient's WBC and NE counts were unstable, even with adenine, cepharanthine, and lithium carbonate treatment. Just before hospitalization to initiate CZP therapy, her WBC and NE levels exceeded the limitations, and her WBC and NE baselines increased. The reason for this is unclear, but one possibility is an increase in body mass index (BMI). Her BMI increased from 19.33 at the first discharge to 21.89 at the second hospitalization. Although there are no reports on the relationship between BMI and WBC and NE levels, patients with anorexia nervosa are known to have low WBC and NE levels.^10^ An increase in BMI may be a solution for low WBC and NE levels during CZP treatment. Exercise and other factors^4^, ^11^, ^12^ temporarily increase NE levels in the bloodstream by mobilizing them from the marginated pool to the circulating pool.^3^ However, we should be careful not to assume that exercise is effective in managing true agranulocytosis, as it never improves hematopoiesis or elevates NE levels. Furthermore, special attention is needed regarding the risk of myocarditis when combining exercise therapy with CZP.^13^ Myocarditis can develop within weeks of starting CZP therapy, so symptoms such as chest pain, shortness of breath, and palpitations should be promptly addressed during exercise.

A limitation of this case report is that we did not evaluate the patient's psychiatric symptoms by any scale and the dosage of CZP was different at each time point. Another limitation is the reference intervals of WBC parameters.^14^ The WBC counts were automatically measured by the same machine. The patient was hospitalized and ate a well‐balanced hospital diet and did not smoke. Blood was collected between breakfast and lunch; however, not at a constant time. This case report does not sufficiently consider the reference intervals for WBC counts. We should corroborate our report with an additional large sample study.

## CONCLUSION

Exercise with subjective and objective evaluation by an OT is effective in increasing WBC or NE counts during CZP for TRS when WBC or NE counts are borderline. If ST exercise is not effective, supervised exercise therapy by an OT is important to continue CZP and may lead to a positive prognosis for patients with TRS.

## AUTHOR CONTRIBUTIONS

**Kenji Hinotsu:** Conceptualization; literature search; data curation; visualization; writing of the original draft. **Shinji Sakamoto:** Conceptualization; project administration; supervision; writing—review and editing. **Hiroki Kawai:** Conceptualization; writing review and editing, visualization. **Yoshio Ohya:** Conceptualization, writing review, and editing. **Takahiro Asada:** Conceptualization, writing review, and editing. **Akiyoshi Yokode:** Conceptualization, writing review, and editing. **Yuko Okahisa:** Conceptualization, writing review, and editing. **Manabu Takaki:** Conceptualization, supervision, writing—review and editing.

## CONFLICT OF INTEREST STATEMENT

Kenji Hinotsu has received research funding from the Kobayashi Magobe Memorial Medical Foundation. Shinji Sakamoto, Hiroki Kawai, Yoshio Ohya, Takahiro Asada, Akiyoshi Yokode, and Yuko Okahisa have no conflicts of interest to declare. Manabu Takaki is an Editorial Board member of *Psychiatry and Clinical Neurosciences Reports* and a coauthor of this article. To minimize bias, he was excluded from all editorial decision‐making related to the acceptance of this article for publication. He reports honoraria from Otsuka, Sumitomo Pharma, Tsumura, Lundbeck, Merck Sharp & Dohme, Eisai, Meiji Seika Pharma, Viatris, Mitsubishi Tanabe Pharma, Janssen, Yoshitomiyakuhin, and Takeda. He has received unrestricted research funding from Otsuka, Sumitomo Pharma, Eisai, and Mochida.

## ETHICS APPROVAL STATEMENT

N/A.

## PATIENT CONSENT STATEMENT

The participant consented to submission of the case report to the journal.

## CLINICAL TRIAL REGISTRATION

N/A.

## Supporting information


**Supplementary Figure: Exercise intensity used in evaluation.** The left side shows the Borg Scale,^6^ which is a subjective evaluation method consisting of a 15‐point scale ranging from 6 to 20 that is used to rate perceived exertion. In our study, verbal confirmation was conducted after exercise. The right side shows the Karvonen formula,^7^ an objective evaluation method using resting heart rate (RR), exercise heart rate (ER), and maximum heart rate (MR). In our report, MR was calculated using the formula “220 − age (years),”^8^ while RR and ER were measured using a pulse oximeter. All values are expressed in beats per minute (bpm).

## Data Availability

The data that support the findings of this study are available on request from the corresponding author. The data are not publicly available due to privacy or ethical restrictions.
