# Supplementary figures and images for: Occupational therapist‐guided exercise increased white blood cell and neutrophil counts during clozapine treatment: A case report
Source: PCN Rep. 2025 Jul 28;4(3):e70167. doi: 10.1002/pcn5.70167 (PMC12303845; doi:10.1002/pcn5.70167)

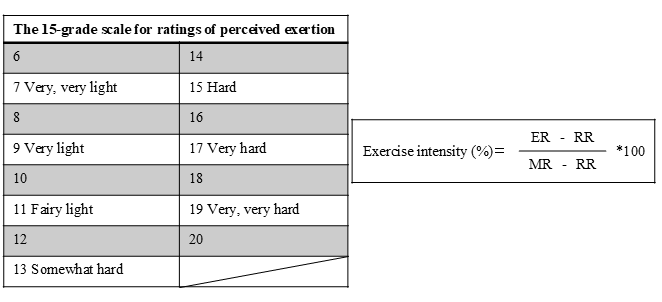

Supplement: Supplementary file 1 — Supplementary Figure: Exercise intensity used in evaluation. The left side shows the Borg Scale,6 which is a subjective evaluation method consisting of a 15‐point scale ranging from 6 to 20 that is used to rate perceived exertion. In our study, verbal confirmation was conducted after exercise. The right side shows the Karvonen formula,7 an objective evaluation method using resting heart rate (RR), exercise heart rate (ER), and maximum heart rate (MR). In our report, MR was calculated using the formula “220 − age (years),”8 while RR and ER were measured using a pulse oximeter. All values are expressed in beats per minute (bpm). [file PCN5-4-e70167-s001.tif]
